# Preclinical safety and biodistribution of SPVN06, a novel gene- and mutation-independent gene therapy for rod-cone dystrophies

**DOI:** 10.1038/s41434-025-00556-3

**Published:** 2025-08-04

**Authors:** Mélanie Marie, Lucie Churet, Anne-Sophie Gautron, Rafal Farjo, Kensuke Mizuyoshi, Victoria Stevenson, Hanen Khabou, Thierry Léveillard, José-Alain Sahel, Florence Lorget

**Affiliations:** 1 SparingVision, Paris, France; 2 EyeCro LLC., Oklahoma City, OK USA; 3 Shin Nippon Biomedical Laboratories (SNBL), Ltd., Kagoshima, Japan; 4 Charles River Laboratories, Mattawan, MI USA; 5 Sorbonne Université, INSERM, CNRS, Institut de la Vision, Paris, France; 6 CHNO des Quinze-Vingts, INSERM-DGOS CIC 1423, Paris, France; 7 Hôpital Fondation Ophtalmologique Adolphe de Rothschild, Paris, France; 8 Department of Ophthalmology, The University of Pittsburgh School of Medicine, Pittsburgh, PA USA

**Keywords:** Gene therapy, Gene expression analysis, Gene delivery, Drug delivery, Diseases

## Abstract

Rod-cone dystrophies (RCD) are caused by mutations in over 100 genes associated with photoreceptor function, leading to progressive and sequential loss of rod and cone photoreceptors. These mutations generally disrupt retinal metabolism and oxidative stress response accelerating disease progression and vision loss. SPVN06 is an adeno-associated virus (AAV)-based gene- and mutation-agnostic investigational therapy designed to slow cone degeneration by delivering long-term expression of rod-derived cone viability factor (RdCVF) and its full-length isoform, thioredoxin RdCVFL, following a single subretinal administration. These proteins support cone survival by promoting glucose metabolism and reducing oxidative damage, respectively, providing a gene and mutation independent therapeutic approach for RCD. SPVN06 IND-enabling program included pharmacology evaluation in the *rd10/rd10* mouse model of RCD (1.0 × 10^8^ vector genomes (vg)/eye up to 1 month) along with systemic and ocular safety and biodistribution evaluation in non-human primates (NHPs, 6.0 × 10^9^ to 3.0 × 10^11^ vg/eye up to 3 months). In the *rd10/rd10* mice, SPVN06 showed preserved vision, as assessed by optokinetic tracking. In NHPs, SPVN06 was well-tolerated up to 6.0 × 10^10^ vg/eye, with high and stable *RdCVF* and *RdCVFL* mRNA expression levels in the retina and retinal pigment epithelium. These results supported the initiation of the ongoing Phase I/II PRODYGY trial with RCD (NCT05748873).

## Introduction

Rod-cone dystrophies (RCD) are a group of rare, inherited retinal diseases characterized by the progressive loss of rod and cone photoreceptors [1, 2]. The most common RCD is retinitis pigmentosa (RP), which affects approximately 1 in 3 to 4000 individuals in the world representing over 1.5 million patients globally [1, 3, 4]. To date, mutations in over 100 genes have been linked to RCD [5], most of which affect genes expressed in rod photoreceptors [2]. Following the initial degeneration of rods, cone photoreceptors subsequently degenerate due to factors including the loss of cone-specific trophic support from rods [6] and increased oxidative stress, as dying rods no longer consume the abundance of oxygen present in the retinal extracellular matrix [7]. This degenerative process generally spans 4–6 decades [1]. Typically, patients initially report a loss of night vision in adolescence, followed by concentric visual field loss in young adulthood, with the loss of central vision occurring later in life, reflecting the sequential degeneration of photoreceptors. Most patients with RCD experience profound visual impairment, with some experiencing total blindness [1]. At present, only one therapy is approved for patients with RCD: voretigene neparvovec-rzyl (Luxturna^®^), which is a gene therapy indicated for the treatment of patients with retinal dystrophies caused by relatively rare *RPE65* mutations [8, 9]. Promisingly, several gene-agnostic therapies (covering retinal cell reprogramming/replacement to immunomodulation, neuroprotection and optogenetics) are now in clinical development, including SPVN06 ([10, 11], https://clinicaltrials.gov/). These gene-agnostic therapies have the potential to treat a broad spectrum of inherited retinal diseases, though the timing and mode of treatment require careful consideration of disease progression and stage. Current gene therapy strategies for RCD aim to replace or correct the causative mutation; however, gene-specific approaches are currently unable to address later disease stages and are only relevant to small patient populations. Given the broad heterogeneity in the genetic mutations that cause RCD, there is a need for gene- and mutation-independent strategies to slow retinal degeneration, regardless of the causative mutation and disease stage [12].

SPVN06 is an adeno-associated virus (AAV)-based gene-agnostic therapy under investigation for the treatment of patients with RCD, independent of the specific gene or mutation involved. Administered via subretinal injection, SPVN06 is designed to promote the long-term production of rod-derived cone viability factor (RdCVF) and its long isoform, RdCVFL. Acting synergistically, RdCVF and RdCVFL aim at slowing or stopping the degeneration of cone photoreceptors, which inevitably leads to blindness in patients with RCD. In healthy retinal cells, endogenous *RdCVF* and *RdCVFL* mRNA are produced by alternative splicing of the nucleoredoxin like 1 (*NXNL1*) gene [13, 14]. Expressed in rod photoreceptors, the trophic factor RdCVF stimulates aerobic glycolysis in cones, increasing the production of carbohydrates essential for cone function and the renewal of cone outer segments [15–17]. RdCVFL, expressed in both rod and cone photoreceptors, is a full-length thioredoxin that protects against oxidative stress by reducing the accumulation of reactive oxygen species [16]. In RCD, due to the degeneration of rods, cone photoreceptors lose their supply of RdCVF, contributing to their subsequent degeneration. Via the production of RdCVF and RdCVFL, SPVN06 induces a synergistic effect through complementary modes of action that support cone photoreceptor survival, leading to the maintenance of visual function, as demonstrated in several proof-of-concept studies [14–16, 18]. By maintaining metabolic and redox pathways, SPVN06 has the potential to slow down the progression of RCD and delay vision loss, regardless of the underlying gene or mutation. Thus, SPVN06 could benefit a significantly larger patient population compared with current gene-specific gene therapy approaches. Importantly, this therapeutic approach could benefit patients at advanced disease stages, when the number of healthy rods is significantly reduced, addressing a critical unmet need where current approaches fall short.

A Phase I/II trial (PRODYGY; NCT05748873) began in early 2023 to assess the safety and tolerability of a single, subretinal injection of SPVN06 in patients with advanced RCD caused by mutations in the *RHO*, *PDE6A*, or *PDE6B* genes. Initial safety and tolerability results have been favorable [19, 20]. This manuscript reports the main preclinical safety and biodistribution findings from studies in non-human primates (NHPs) that enabled the initiation of the ongoing clinical investigation of SPVN06. To support the rationale for clinical dose selection, the pharmacology of SPNV06 in a *rd10/rd10* mouse model of autosomal recessive RP (and RCD per extension) is also briefly described [21, 22].

## Materials and Methods

### SPVN06 vector construct

**Fig. 1:**
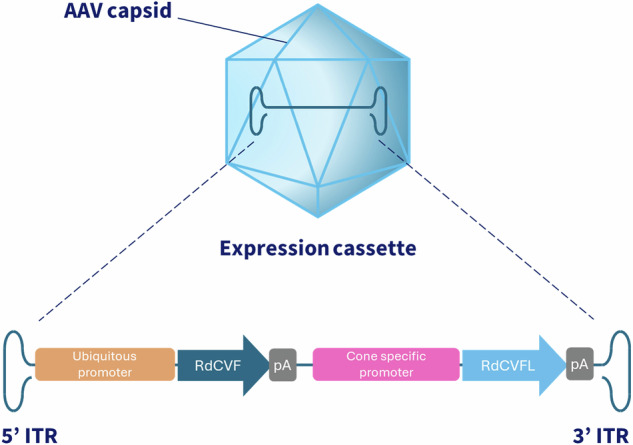
SPVN06 structure. SPVN06 encodes the two synergistic isoforms of *NXNL1*, *RdCVF* and *RdCVFL*, in a single vector. The total size of the expression cassette is 4926 base pairs. AAV adeno-associated virus, ITR inverted terminal repeats, RdCVF rod-derived cone viability factor, RdCVFL rod-derived cone viability factor long-form, pA polyadenylation regulatory sequences.

SPVN06 is a recombinant AAV vector, with a serotype 8 capsid that encodes the two synergistic isoforms of *NXNL1*, *RdCVF* and *RdCVFL*, in a single vector genome (Fig. 1). Flanked by AAV serotype 2 inverted terminal repeats, the expression cassette (4926 base pairs) contains two codon-optimized cDNAs: one encoding RdCVF under the control of the ubiquitous promoter cytomegalovirus/chicken β-actin (CMV/CBA), and the other encoding RdCVFL under the control of the cone-specific L-opsin promoter PR1.7. SPVN06 was manufactured using clinically relevant process. The GMP clinical batch was used in the GLP low dose study.

### Animal procedures

#### rd10/rd10 mice

Studies in *rd10/rd10* mice were conducted at the EyeCRO animal facility (accredited by the American Association for Accreditation of Laboratory Animal Care [AAALAC] International). All experimental procedures adhered to the Association for Research in Vision and Ophthalmology [ARVO] Statement for the Use of Animals in Ophthalmic and Vision Research and were approved by the EyeCRO Institutional Animal Care and Use Committee (approval number SOW003 IACUC #2019-01-17-002). Data from a separate study conducted at the same facility in naïve wild-type (WT) mice were included to serve as an additional reference point (approval number SOW001 IACUC #2019-01-17-002).

WT C57BL/6 J and *rd10/rd10* mice [23] were bred in-house. Both males and females were included, with a minimum of five animals per gender and per group at the final necropsy timepoint. Study design including dosing groups and animal numbers is presented in Table 1 and was done in accordance with regulatory guidelines requirements. To prevent premature phototoxicity-induced retinal degeneration and to limit inter-individual variability, *rd10/rd10* mice were raised in darkness from birth to postnatal day (P) 30 and transferred to a 12 h light/dark cycle on P31. Animals were housed in the lower racks of the caging system to minimize exposure to light, with ~200 lux environmental illumination and ~40 lux in cages from P31 to study termination. WT mice were raised and maintained on a normal light/dark cycle from birth to final necropsy. Animals had access to food and water *ad libitum*. Animals were scheduled to be terminated on P48, at which point samples were collected for the evaluation of DNA/mRNA expression. Mice did not receive any immunosuppressive regimen.

**Table 1:** Study design in *rd10/rd10* mice.

*Strain*	*Treatment*	*Dose*		*Number and gender*
		*Injection volume (µl)*	*Total dose (vg/eye)*	
WT C57BL/6J^a^	No treatment	N/A	0	5 M, 5 F
*rd10/rd10*	Vehicle (OU)	1	0	7 M, 7 F
	SPVN06 (OU)	1	1.0 × 10^8^	6 M, 6 F

^a^Data from a separate study performed in the same animal facility in WT mice were included to provide an additional reference point.

All injections were OU; both eyes were treated and received one subretinal administration of either vehicle or SPVN06. The SPVN06 dose of 1.0 × 10^8^ vg/eye was selected based on previous studies conducted with either SPVN06 or its prototypes at various doses.

*F* female, *M* male, *N/A* not applicable, *OU* oculus uterque, *vg* vector genomes, *WT* wild-type.

#### Non-human primates

Three studies were conducted in NHPs: one 4-week pilot study, and two 13-week Good Laboratory Practice (GLP) studies: a high-dose study and a low-dose study, each involving multiple doses. All three studies were conducted with cynomolgus macaques (*Macaca fascicularis*). The 4-week pilot study and GLP high-dose study were conducted at the Charles River Laboratories in Mattawan, USA (CR-MWN), where protocols were reviewed and approved by the CR-MWN Institutional Animal Care and Use Committee (IACUC, ethical body) in accordance with the guidelines of the US National Research Council (approval numbers: 3228-001 for the pilot study and 3228-002 for the GLP high-dose study). A second GLP study using lower doses of SPVN06 (GLP low-dose study) was conducted subsequently at Shin Nippon Biomedical Laboratories, Ltd (SNBL) in Kagoshima, Japan. The protocol for this study was reviewed and approved by SNBL IACUC (approval number IACUC563-001) and Recombinant Gene Experiment Safety Committee (approval number LMOD-563001), and was conducted in compliance with the animal welfare regulations of SNBL, which is accredited by AAALAC International.

The 4-week pilot study was conducted in four female animals (age range: 2 − 3 years; weight range: 2.0 − 2.2 kg), while the two 13-week GLP studies were conducted in at least three animals per gender per dose group (age range: 2 − 4 years; weight range: 1.6 − 4.8 kg). Study designs including dosing groups and animal numbers is presented in Table 2 and was done in accordance with regulatory guidelines requirements.

**Table 2:** Study design in NHPs.

Study duration, GLP status	Treatment (OU)	Dose		Number and gender	
		Injection volume (µl)	Total dose (vg/eye)	Terminated at 4 weeks^a^	Terminated at 13 weeks^b^
4-week, non-GLP pilot study	Vehicle	100	0	1 F	0
	SPVN06	100	7.0 × 10^10^	3 F	0
13-week, GLP low-dose study	Vehicle	100	0	3 M, 3 F	3 M, 3 F
	SPVN06	100	6.0 × 10^9^	0	3 M, 3 F
	SPVN06	100	2.0 × 10^10^	0	3 M, 3 F
	SPVN06	100	6.0 × 10^10^	3 M, 3 F	3 M, 3 F
13-week, GLP high-dose study	Vehicle	100	0	0	3 M, 3 F
	Vehicle	150	0	1 M, 1 F	3 M, 3 F
	SPVN06	100	1.0 × 10^11^	0	3 M, 3 F
	SPVN06	100	2.0 × 10^11^	0	3 M, 3 F
	SPVN06	150	3.0 × 10^11^	2 M, 2 F	3 M, 3 F

^a^Termination was on Day 30 for the 4-week non-GLP pilot study and on Day 28 for the 13-week GLP studies.

^b^Day 91.

All injections were OU; both eyes were treated and received one subretinal administration of either vehicle or SPVN06.

*F* female, *GLP* Good Laboratory Practices, *M* male, *OU* oculus uterque, *vg* vector genomes.

A 12-hour light/dark cycle was maintained in the animal facility. Food was provided daily according to the animal facility’s standard operating procedure, with water available *ad libitum*. Across studies, all animals received a standard immunosuppressive regimen, including intramuscular (IM) methylprednisolone at 40 mg on the day prior to dosing and weekly for 4 weeks thereafter, to mitigate potential immune reactions to the capsid and/or transgene. Animals were scheduled to be terminated at Weeks 4 or 13 post-injection, at which point samples were collected for the evaluation of safety and biodistribution.

### Anesthesia

#### rd10/rd10 mice

Prior to the subretinal injection of SPVN06, *rd10/rd10* mice were anesthetized via intraperitoneal injection of 85 mg/kg ketamine and 14 mg/kg xylazine.

#### Non-human primates

Prior to the subretinal injection of SPVN06, NHPs were anesthetized using either ketamine (7 mg/kg, IM) and isoflurane (to effect, inhalation), or ketamine (10 mg/kg, IM) and xylazine in a 7:1 ratio (v/v). For ocular examinations (including optical coherence tomography [OCT], indirect ophthalmoscopy, slit-lamp and eye fundus imaging, and electroretinography), anesthesia was induced and maintained using either dexmedetomidine (0.01 − 0.02 mg/kg, IM) and ketamine (5 − 15 mg/kg, IM), or ketamine alone (10 mg/kg). Anesthesia for termination was conducted according to the standard operating procedure of each animal facility, using either ketamine (5 − 20 mg/kg, IM), or ketamine (50 mg/kg, IM) and medetomidine (0.08 ml/kg).

### Subretinal injection procedures

All subretinal injections were administered as a single dose in both eyes. Animals were either injected with SPVN06 or vehicle. Vehicle injections contained SPVN06 final formulation buffer (sterile balanced saline solution, supplemented with 0.001% Kolliphor P188^®^, pH 7.2).

#### Rd10/rd10 mice

Injections of either vehicle or SPVN06 were administered on P18 in a dark room under a dim red light. Mouse eyes were dilated, and animals were anesthetized as previously described before being placed on a regulated heating pad. The posterior pole was visualized under magnification. A 12.7 mm, 30-gauge insulin syringe was used to puncture the cornea just above the corneal limbus, avoiding any contact with the sclera and lens. SPVN06 or vehicle was delivered into the subretinal space using a 10 μl Hamilton syringe with a 33-gauge blunt needle, which was inserted through the corneal puncture across the vitreous, with the shaft aimed at the back of the eyecup, avoiding any trauma to the lens or iris. Injection volume and dosing are described in Table 1. Immediately following administration, the retina was examined using high-magnification microscopy to verify the presence of a well-formed bleb and the absence of unexpected events, such as retinal detachment outside the injection area or retinal hemorrhage.

#### Non-human primates

Animals were anesthetized as previously described and positioned in dorsal recumbency. The eyes and eyelids were disinfected and maintained open using a sterilized blepharostat. A microvitreoretinal blade with a 25-gauge valved cannula was inserted through the conjunctiva and sclera and directed into the vitreous humor. The trocar was positioned to face the posterior axis of the globe before being retracted, leaving the scleral port in place. A direct contact surgical lens was placed on the cornea using sterile coupling gel. An endoilluminator probe was inserted through one of the scleral ports to enable direct visualization of the posterior segment through the microscope. Next, a small-diameter, subretinal injection cannula was inserted through a second port and advanced into the mid-vitreous until it made contact with the retinal surface. The dosing solution was then administered slowly, inducing and filling a subretinal bleb. If appropriate bleb formation was observed, the injection was continued to deliver the entire dose volume (100 or 150 μl) into the subretinal space. SPVN06 doses ranged from 6.0 × 10^9^ to 3.0 × 10^11^ vector genomes (vg)/eye (Table 2). After delivering the dose, the injection cannula and endoilluminator probe were removed from the scleral ports, and the contact lens from the cornea. The scleral ports were then removed, and the lateral canthotomy site was closed using a 7-0 Vicryl suture.

### Optokinetic tracking (OKT)

Mouse visual function was measured on P32, P38, and P45 using a virtual OKT system designed for rodent use (OptoMetry, Cerebral Mechanics Inc. Medicine Hat, Alberta, Canada) as previously described [24]. Briefly, mice were placed on a platform surrounded by four screens displaying continuous vertical sine wave gratings that rotated, creating the illusion of a 3D rotating cylinder. Visual performance was assessed by the mouse’s ability to track the rotating grating with its head. Spatial frequencies ranging from 0.064 to 0.514 cycles/degree were tested to establish the spatial frequency threshold, with each eye tested independently. No tracking behavior in response to stimuli was associated with a ‘0’ spatial frequency threshold.

### Full-field electroretinography (ffERG)

To assess cone function in NHPs, ffERG was conducted pre-injection, and at Weeks 4 and 13 using an electroretinoscope (Ganzfeld System SG-2002, LKC Technologies, Inc., Gaithersburg, MD, USA or Retiport Gamma, Roland Consult, Brandenburg an der Havel, Germany). Prior to testing, NHPs were anesthetized as previously described and dark-adapted for 30 − 60 min. Once pupil dilation was confirmed, a surface anesthetic and mucous membrane-protecting agent were instilled into the eye. A contact lens-type electrode for primates was then attached to the cornea. After the electric potential stabilized, ffERG examinations were conducted to measure cone response using a luminescence intensity light of 0 dB (3.0 cds/m^2^). The resulting tracings were used to measure b-wave values.

### Ocular inflammation scoring

NHP eyes were assessed for aqueous and vitreous cells using a hand-held slit-lamp biomicroscope to evaluate ocular inflammation. Assessments were conducted pre-injection and on Days 7, 14, 28, 42, and 85 post-injection for the GLP high-dose study, conducted at Charles River Laboratories, with additional timepoints of Days 3, 61, and 91 post-dosing evaluated in the GLP low-dose study at SNBL. Each site employed its own, but comparable, standard grading system for the number of cells observed in the examination field, based on the semiquantitative preclinical ocular toxicology scoring system (SPOTS) [25].

### OCT, anterior segment and eye fundus imaging

OCT was conducted in NHPs under full sedation at baseline, immediately after subretinal injection, and at Weeks 4 and 13 post-injection to evaluate bleb formation and retinal integrity (Heidelberg Spectralis OCT2 system, Heidelberg Engineering GmbH, Heidelberg, Germany). Additionally, a RetCam Shuttle Digital Imaging system (Clarity MSI, Pleasanton, CA, USA) was used to capture digital color images at baseline, Day 1, Week 4 and Week 13 post-injection, allowing for observation of the ocular fundus, lens and anterior segment structures.

### Biodistribution and transgene mRNA expression

#### Mouse tissue collection

Following necropsy on P48, mouse eyes were enucleated and retinas were dissected and snap-frozen in liquid nitrogen for DNA and mRNA extraction.

#### NHP tissue collection

Fluids were collected at multiple timepoints during the study and immediately snap-frozen in liquid nitrogen. Following necropsy at either Week 4 or Week 13 post-injection, NHP eyes were enucleated. One eye was dedicated to histology analysis and the other eye to biodistribution analysis. For biodistribution analysis (genomic), ocular and systemic tissues were dissected individually before being snap-frozen in liquid nitrogen. During dissection of the eye, the retinal bleb area (6 − 8 mm) was isolated from the remaining retina (outside of the bleb area). Care was taken during sample collection to avoid cross-contamination between tissues. Sentinels were included in the extraction process to detect any potential cross-contamination between samples.

The tissues and fluids collected for qPCR (quantitative polymerase chain reaction) analysis included the retina bleb and remaining retina, aqueous humor, vitreous humor, combined bulbar and palpebral conjunctiva, combined choroid and retinal pigment epithelium (RPE), cornea, iris/ciliary body, lens, sclera bleb and remaining sclera, optic nerve, optic chiasm, optic tract, visual cortex, lateral geniculate nucleus, cerebellum, superior colliculus, frontal cortex, mandibular lymph node, spleen, heart, kidney, liver (caudate, lateral left, lateral right, median), lung, ovary, testis, whole blood (collected pre-injection and on Days 1, 4, 7, 14, 26 or 28, 60 or 62, 90) and tears (collected pre-injection and on Days 1, 4, 7, 14, 26 or 28, 60 or 62, 90). RT (reverse transcription)-qPCR analysis was performed on the retinal bleb and remaining retina.

#### DNA extraction and qPCR

Total DNA was extracted from mouse retinas and NHP tissues using a Nucleospin^®^ Tissue kit (Macherey-Nagel, Düren, Germany). For NHP whole blood, DNA was extracted using a NucleoSpin^®^ Blood QuickPure kit (Macherey-Nagel, Düren, Germany), and for NHP tears, a QIAamp Viral kit was used (Qiagen, Hilden, Germany). The concentration and purity of the extracted DNA were determined by ultraviolet spectrophotometry using a Nanodrop 8000 (Thermo Fisher Scientific, Waltham, MA, USA) for all samples, except fluids. Purity was assessed using absorbance ratios of OD_260/280 nm_ and OD_260/230 nm_.

PCR was performed using specific primers and double-quenched probes labeled with a fluorescein amidites (FAM) reporter dye (ZEN/Iowa Black™ fluorescence quencher, Integrated DNA Technologies, Leuven, Belgium). Reactions were conducted at a final primer and probe concentration of 200 nM. Each qPCR reaction mix had a total volume of 50 μl, comprising 10 μl of the DNA sample and 40 μL TaqMan™ Gene Expression Master Mix (Applied Biosystems, Foster City, CA, USA). Plates were run on a QuantStudio™ 7 PCR system (Applied Biosystems, Foster City, CA, USA) using the following protocol: one cycle at 50 °C for 2 min and 95 °C for 10 minutes; 40 cycles at 95 °C for 15 s and 62 °C for 1 min.

#### RNA extraction and RT-qPCR

Total RNA was extracted from NHP and mouse retinas using a RNeasy Mini Kit (Qiagen, Hilden, Germany). The concentration and purity of the extracted RNA were determined by ultraviolet spectrophotometry using a Nanodrop 8000 (Thermo Fisher Scientific, Waltham, MA, USA) for all samples. Purity was assessed using absorbance ratios of OD_260/280 nm_ and OD_260/230 nm_. The primers and probes for *RdCVF* and *RdCVFL* mRNA quantification (Integrated DNA Technologies, Leuven, Belgium) were designed to target the exogenous *RdCVF* and *RdCVFL* mRNA produced upon SPVN06 injection to distinguish them from homologous endogenous mRNA. Each probe was 5’-labeled with a FAM reporter dye and 3’-conjugated with a non-fluorescent quencher. Reactions were conducted at a final primer and probe concentration of 400 nM. The RT-qPCR reaction mixes had a total volume of 20 μl, containing 5 μL of RNA sample and 15 μL TaqMan™ Fast virus 1-Step Master Mix for qPCR (Applied Biosystems, Foster City, CA, USA). Plates were run on a QuantStudio™ 7 PCR System (Applied Biosystems, Foster City, CA, USA) using the following protocol: one cycle at 50 °C for 5 minutes and 95 °C for 20 s; 40 cycles at 95 °C for 3 seconds and 62 °C for 30 s.

#### qPCR and RT-qPCR analysis

DNA and RNA samples were analyzed alongside a calibration curve consisting of eight calibration standards and two sets of quality control samples. Calibration standards were prepared using linearized plasmid SPVN06 (pSPVN06) in 1.00 µg of herring sperm DNA (surrogate DNA) for qPCR, or *RdCVF* and *RdCVFL* RNA in 100 ng of yeast RNA (surrogate RNA) for RT-qPCR.

The corresponding Ct values were plotted against the base 10 logarithm of the calibration standard copy number and a linear curve was fitted using Microsoft Excel. SPVN06 DNA or *RdCVF* and *RdCVFL* mRNA copy numbers in each sample were then interpolated from the calibration curve. SPVN06 DNA (vector genome) quantity and *RdCVF* and *RdCVFL* mRNA quantities are reported as absolute quantifications per µg of DNA or RNA.

#### In situ hybridization

A BaseScope™ Duplex Assay (ACDbio, Newark, CA USA) was performed on whole eye sections from NHPs enrolled in the 4-week pilot study to visualize the distribution of SPVN06 DNA alongside *RdCVF* and *RdCVFL* transgene mRNA expression. Following necropsy, eyes were preserved in Davidson’s fixative, paraffin-embedded, and prepared according to laboratory guidelines. Pre-treatment conditions for the Mild BaseScope™ Duplex Assay included target retrieval for 15 min at 95 − 100 °C followed by incubation with protease IV for 15 min at 40 °C. A custom pigment bleaching step was performed on all tissue samples prior to staining. Specific probes were designed to detect *RdCVF* mRNA (blue signal) and *RdCVFL* mRNA (pink signal). Both probes were also able to detect SPVN06 DNA.

### Histology

Unblinded qualitative histological analyses were conducted by board-certified pathologists to assess retinal structure and evaluate any microscopic changes associated with the subretinal injection of SPVN06 compared with vehicle-treated eyes. NHPs were terminated as previously described at either Week 4 or 13 post-injection. After enucleation, eyes were fixed and sectioned into two parts with a single cut superior to the optic nerve, ensuring that both the optic disc and fovea were included. Eyes were then paraffin-embedded and trimmed. Slides were either stained with hematoxylin and eosin (H&E) or incubated with anti-cone arrestin (LS-C368677, LifeSpan Bioscience Inc., Seattle, WA, USA) or anti-RPE65 (NB100-355, NOVUS Biologicals, LLC, Centennial, CO, USA) antibodies.

### Immunogenicity analysis

The immunogenicity of SPVN06 in NHPs was evaluated using an electrochemiluminescent bridging immunoassay designed to detect total anti-AAV8 antibodies in primate serum following SPVN06 administration. Samples were collected pre-injection and on Days 1 (30 min post-dose; relative to the second eye dosed), 4, 7, 14, 30, 60 and 90 (prior to necropsy).

Briefly, a Meso Scale Discovery (MSD) plate was coated with unlabeled AAV8 overnight. The following day, the MSD plate was washed with 0.05% Tween-20 in 1 x phosphate buffered saline (PBS), and subsequently blocked with assay buffer (casein in PBS). Samples were diluted 1:10 in assay buffer and incubated in the MSD plate. Following incubation, the plate was washed, and ruthenylated AAV8 was added. After a final wash, MSD read buffer was added and the plate was read on an MSD reader (Mesoscale Discover S600, Mesoscale Diagnostics, Rockville, MD, USA).

The presence of total anti-AAV8 antibodies was determined by comparing the sample signals to the assay cut point, with samples showing a mean signal equal to or greater than this threshold considered positive. Titer was determined for positive samples.

### Statistical analysis

Animals (mice and primates) were randomly assigned to study groups; analyses were not performed in a blinded fashion. Each eye was treated as an individual data point, as subretinal administration was performed independently in both eyes for all animals. Data from mouse eyes with procedure-related defects that impaired proper ocular functional assessments including ocular atrophy, corneal defects, cataracts, or retinal hemorrhages that cover >50% of the retina in fundus images were excluded from the functional data sets. No data was excluded from the non-human primate studies. While many of the findings were qualitative in nature, statistical analyses were conducted where possible and relevant, using one- or two-way ANOVA with Tukey’s post-hoc test to compare treatment and dose groups, with analyses performed using GraphPad Prism software (version 9.3.0, GraphPad Prism, Boston, MA, USA). A p-value < 0.05 was considered statistically significant.

## Results

### SPVN06 pharmacological activity in the rd10/rd10 mouse model of retinitis pigmentosa

**Fig. 2:**
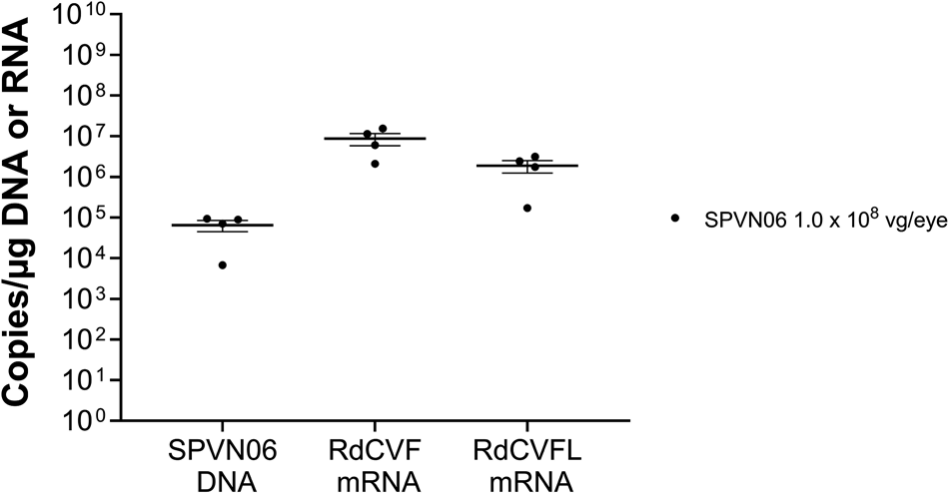
SPVN06 DNA and *RdCVF* and *RdCVFL* mRNA expression in the retina of *rd10/rd10* mice following SPVN06 subretinal bilateral injection. Quantification of SPVN06 DNA (vector genome copies, qPCR) and *RdCVF* and *RdCVFL* transgene mRNA expression (RT-qPCR) in the mouse retina at Week 4 post-injection with SPVN06 at 1.0 × 10^8^ vg/eye (*N* = 4 eyes). Data shown are mean ± SEM, with each point representing an individual retinal sample. qPCR quantitative polymerase chain reaction, RdCVF rod-derived cone viability factor, RdCVFL rod-derived cone viability factor long-form, RT-qPCR quantitative reverse transcription polymerase chain reaction.

To prevent premature phototoxicity-induced retinal degeneration and to limit inter-individual variability, *rd10/rd10* mice were raised in darkness from birth, and transferred to a 12-hour light/dark cycle on P31. The characterization of this mouse model has been described by Marie et al. [22]. SPVN06 was administered subretinally on P18 under a dim red light. SPVN06 DNA, and *RdCVF* and *RdCVFL* transgene mRNA expression were quantified in all retinal samples at Week 4 post-injection of SPVN06 at 1.0 × 10^8^ vg/eye (qPCR and RT-qPCR analyses) (Fig. 2). SPVN06 DNA was quantified at a mean ± SEM of 6.46 × 10^4^ ± 1.99 × 10^4^ copies/µg DNA, *RdCVF* mRNA at a mean of 8.7 × 10^6^ ± 2.91 × 10^6^ copies/µg RNA and *RdCVFL* mRNA at a mean of 1.87 × 10^6^ ± 6.33 × 10^5^ copies/µg RNA. No gender differences were observed. SPVN06 DNA and *RdCVF* and *RdCVFL* mRNA levels were below the limit of detection in the retinal samples from Vehicle-treated animals.

**Fig. 3:**
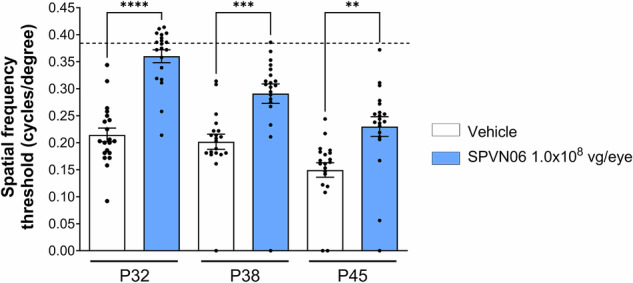
Visual function in *rd10/rd10* mice following SPVN06 subretinal bilateral injection. OKT was used to evaluate visual function on P32, P38, and P45 in *rd10/rd10* mice treated with SPVN06 1.0 × 10^8^ vg/eye compared with vehicle-treated mice. The dotted line represents WT visual function on P38, based on data from a separate study. Data shown are mean ± SEM, with each point representing an individual eye (N = 20 eyes/group). Statistical analysis was performed using a one-way ANOVA (Tukey’s post-test), where **p ≤ 0.01, ***p ≤ 0.001, and ****p ≤ 0.0001. OKT optokinetic tracking, P postnatal day, WT wild-type.

To characterize the pharmacological activity of SPVN06 in dark-reared *rd10/rd10* mice, visual function was assessed using OKT starting 15 days post-injection once animals had been transferred to light conditions (Fig. 3). Compared with vehicle-treated mice, those treated with SPVN06 at 1.0 × 10^8^ vg/eye exhibited significantly higher visual function at all evaluated time points (Fig. 3). On P32, visual function in SPVN06-treated eyes was comparable to WT levels, while in vehicle-injected eyes, it was reduced to approximately half of WT levels. There was no significant slowing of visual function loss measured by ffERG (Supplementary File 2A–C) nor was there any obvious structural protection of the mouse retina on the OCT imaging measurements (Supplementary File 2D, E). Illustrative images of hematoxylin and eosin histology (P48) revealed comparable retinal anatomy with one row of cone nuclei (visualized by cone arrestin labeling) in the ONL in both vehicle and 1.0 × 10^8^ vg/eye SPVN06-treated retina (Supplementary File 2F, G). Based on the OKT results, 1.0 × 10^8^ vg/eye was carried forward as the pharmacologically active dose of SPVN06.

### SPVN06 distribution in the retina of NHPs

**Fig. 4:**
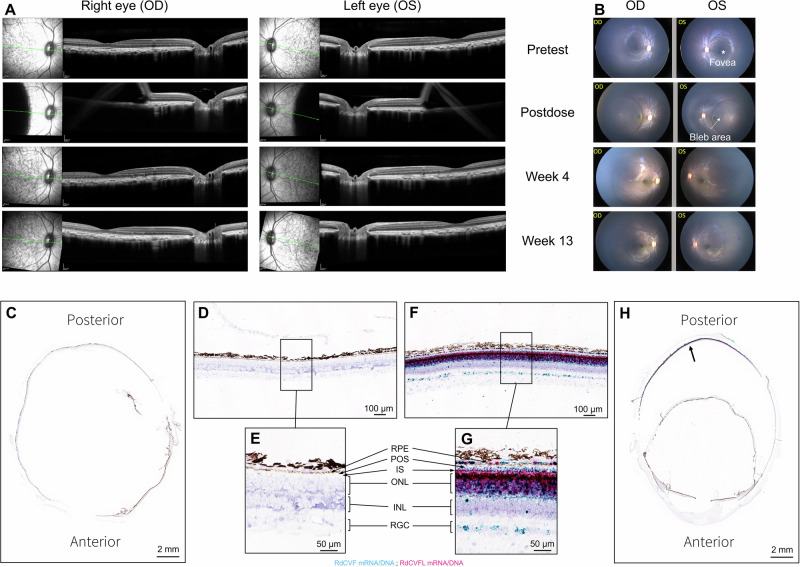
Bleb formation and SPVN06 distribution in NHPs following SPVN06 subretinal bilateral injection. Representative OCT (**A**) and eye fundus (**B**) images of NHP eyes before subretinal injection, immediately after injection, and at Weeks 4 and 13 post-injection. In (**B**), the star indicates the fovea location, and the white arrow the bleb location. **C**, **H** Low magnification of *RdCVF* and *RdCVFL* DNA/mRNA expression in the NHP eye evaluated in the 4-week pilot study using in situ hybridization at Week 4 post-injection with either vehicle (**C**) or SPVN06 at 7.0 × 10^10^ vg/eye (**H**, black arrow). High-magnification in situ hybridization focused on the retina of vehicle-treated (**D**, **E**) and SPVN06-treated (**F**, **G**) eyes. Probes targeting *RdCVF* (blue signal) and *RdCVFL* (pink signal) are shown. Images shown are representative of one animal for each treatment group. IS photoreceptor inner segments, NHP non-human primate, OCT optical coherence tomography, OD oculus dexter, ONL outer nuclear layer, INL inner nuclear layer, OS oculus sinister, POS photoreceptor outer segments, RGC retinal ganglion cells, RPE retinal pigment epithelium.

In NHPs, bleb formation was assessed following bilateral subretinal injection of SPVN06 or vehicle using OCT (Fig. 4A) and eye fundus imaging (Fig. 4B). Retinal re-attachment was confirmed at Weeks 4 and 13 post-injection. In the 4-week pilot study, the distribution of SPVN06 DNA and transgene mRNA were assessed to characterize SPVN06 spread from the injected bleb to the entire retina. In situ hybridization was performed on NHP whole eye sections at Week 4 post-injection with SPVN06 at 7.0 × 10^10^ vg/eye. The results showed that the spread of SPVN06 vector DNA and transgene mRNA expression was larger than the bleb area and covered most of the retina (Fig. 4H). No SPVN06 DNA or transgene mRNA was observed following vehicle injection (Fig. 4C). When viewed at higher magnification, SPVN06 DNA and *RdCVF* and *RdCVFL* mRNA expression were highest in the photoreceptor layer and in RPE cells (Fig. 4D–F). *RdCVF* and *RdCVFL* mRNA expression were also observed in other retinal layers, including the inner nuclear and ganglion cell layers. No expression was detected in vehicle-treated eyes (Fig. 4E).

### Genomic analysis of SPVN06 biodistribution and shedding in NHPs

**Fig. 5:**
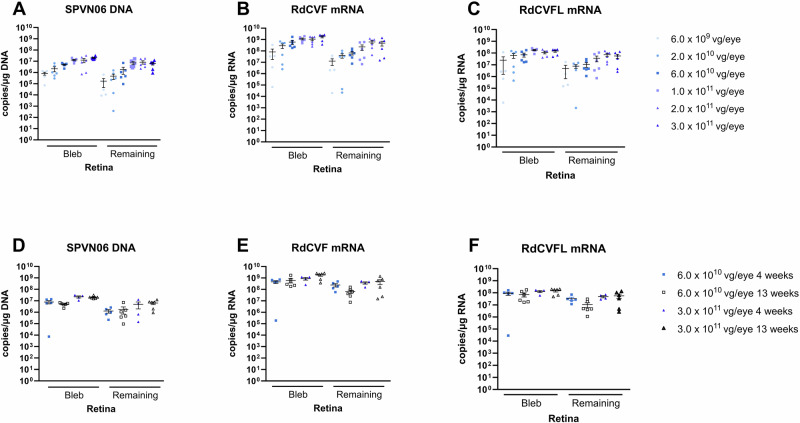
SPVN06 DNA and *RdCVF* and *RdCVFL* mRNA expression in the retina of NHPs following SPVN06 subretinal bilateral injection. Quantification of (**A**) SPVN06 DNA (vector genome copies, qPCR) and (**B**) *RdCVF* and (**C**) *RdCVFL* mRNA expression (RT-qPCR) in the retinal bleb and remaining retina at Week 13 post-injection with all doses of SPVN06 (N = 6 eyes). Quantification of (**D**) SPVN06 DNA (vector genome copies, qPCR) and (**E**) *RdCVF* and (**F**) *RdCVFL* mRNA expression (RT-qPCR) in the retinal bleb and remaining retina at Weeks 4 and 13 post-injection with SPVN06 at 6.0 × 10^10^ and 3.0 × 10^11^ vg/eye (N ≥ 4 eyes). Data shown are mean ± SEM, with each point representing an individual eye. NHP, non-human primate; qPCR, quantitative polymerase chain reaction; RdCVF, rod-derived cone viability factor; RdCVFL, rod-derived cone viability factor long-form; RT-qPCR, quantitative reverse transcription polymerase chain reaction.

In the two GLP studies, SPVN06 DNA and *RdCVF* and *RdCVFL* mRNA in the retinal bleb and the remaining retina (outside the bleb area) were quantified using qPCR and RT-qPCR analyses (Fig. 5). SPVN06 DNA and transgene mRNA distribution were not limited to the retinal bleb, as DNA/mRNA were also quantified in the remaining retina at Weeks 4 and 13 post-injection, although levels were lower ( ~ 1 log), confirming the in situ hybridization data from the 4-week pilot study. At Week 13 post-injection with SPVN06, quantification of SPVN06 DNA, *RdCVF* and *RdCVFL* mRNA expression levels in the retinal bleb and remaining retina showed a dose-dependent trend toward increased expression at higher SPVN06 doses (Fig. 5A, B, D, E); conversely, *RdCVFL* mRNA expression seemed to reach a plateau at doses ≥ 1.0 × 10^11^ vg/eye (Fig. 5C, F). SPVN06 DNA, *RdCVF* and *RdCVFL* mRNA expression levels were comparable between Week 4 and 13 post-injection (Fig. 5D–F).

Additionally, SPVN06 biodistribution and shedding were evaluated across a comprehensive range of ocular, brain, and systemic tissues in the GLP low-dose study (Table 3). Across the examined doses, analyses confirmed that SPVN06 DNA was not restricted to the retinal bleb; DNA was also quantified in other retinal and ocular regions up to Week 13 post-injection, including the choroid/RPE, sclera, iris/ciliary body, lens, and cornea. However, SPVN06 DNA levels in these regions were generally lower than in the retinal bleb. Low levels of SPVN06 DNA were also found in non-ocular tissues, with the highest quantities detected in the spleen and lymph nodes as anticipated for this capsid and route of administration [26]. No corresponding transgene mRNA expression was detected in these tissues. No SPVN06 DNA was detected in the reproductive organs, heart, frontal and visual cortices, or cerebellum, with no gender differences observed. Additionally, no SPVN06 DNA was detected in vehicle-treated animal tissues, confirming the specificity of the genomic methods used (data not shown).

**Table 3:** Quantification of SPVN06 DNA in NHP tissue at Weeks 4 and 13 after SPVN06 subretinal bilateral injection.

SPVN06 DNA (copies/µg DNA)	Week 4			Week 13								
	6.0 × 10^10^ vg/eye			6.0 × 10^9^ vg/eye			2.0 × 10^10^ vg/eye			6.0 × 10^10^ vg/eye		
	BLD^a^	BLQ^b^	Mean Copy^c^	BLD^a^	BLQ^b^	Mean Copy^c^	BLD^a^	BLQ^b^	Mean Copy^c^	BLD^a^	BLQ^b^	Mean Copy^c^
*Ocular Tissue*												
Retina left bleb			5 (7.87 × 10^6^)			5 (8.24 × 10^5^)			6 (2.17 × 10^6^)			6 (5.02 × 10^6^)
Retina left remaining			6 (1.26 × 10^6^)			5 (1.69 × 10^5^)			6 (4.78 × 10^5^)			6 (1.79 × 10^6^)
Aqueous humor	4	1		5			6			6		
Vitreous humor			5 (1.11 × 10^2^)	4	1		3	2	1 (1.32 × 10^1^)	3	2	1 (1.63 × 10^1^)
Bulbar and palpebral conjunctiva	1		4 (1.48 × 10^3^)	4	1		3		3 (3.02 × 10^3^)		1	5 (2.52 × 10^3^)
Choroid/RPE bleb			5 (1.10 × 10^7^)			5 (1.32 × 10^6^)			6 (3.91 × 10^6^)			6 (7.46 × 10^6^)
Choroid/RPE remaining			5 (1.85 × 10^6^)			5 (2.10 × 10^5^)			6 (1.25 × 10^6^)			6 (2.39 × 10^6^)
Cornea			5 (1.44 × 10^4^)		3	2 (4.05 × 10^2^)			6 (7.45 × 10^3^)			6 (5.76 × 10^3^)
Iris/Ciliary body			5 (1.68 × 10^5^)			5 (5.73 × 10^3^)			6 (8.17 × 10^3^)			6 (1.09 × 10^4^)
Lens^d^			5 (4.15 × 10^4^)		1	4 (1.19 × 10^4^)		1	5 (2.39 × 10^5^)			6 (3.11 × 10^4^)
Sclera			5 (3.86 × 10^5^)			5 (2.83 × 10^4^)			6 (4.43 × 10^5^)			6 (4.84 × 10^5^)
Optic nerve			5 (4.51 × 10^4^)			5 (1.02 × 10^3^)			6 (3.83 × 10^3^)			6 (3.29 × 10^4^)
Optic chiasm			6 (1.07 × 10^4^)		1	4 (1.02 × 10^3^)			6 (4.91 × 10^2^)			6 (1.36 × 10^3^)
Optic tract			6 (4.02 × 10^3^)		1	5 (1.81 × 10^3^)			6 (8.07 × 10^2^)			6 (2.32 × 10^3^)
Visual cortex	6			6			6			6		
LGN			6 (7.66 × 10^2^)	2		4 (1.16 × 10^3^)		1	5 (2.69 × 10^2^)			6 (5.95 × 10^2^)
Cerebellum	6			6			6			6		
Superior colliculus	1	3	2 (9.98 × 10^1^)	2	3	1 (5.63 × 10^2^)	2	1	3 (1.66 × 10^2^)		2	4 (1.67 × 10^2^)
Frontal cortex	3	3		6			6			4	2	
Mandibular lymph nodes		1	5 (3.91 × 10^4^)	2	2	2 (5.23 × 10^2^)		2	4 (3.73 × 10^3^)		1	5 (3.39 × 10^4^)
Spleen			6 (2.18 × 10^4^)		2	4 (5.33 × 10^2^)			6 (2.24 × 10^3^)		2	4 (6.12 × 10^3^)
Heart	6			6			6			6		
Kidney	5		1 (1.02 × 10^2^)	6			6			6		
Liver caudate	5		1 (7.39 × 10^2^)	6			6			5		1 (1.23 × 10^2^)
Liver lateral left	2	3	1 (7.19 × 10^2^)	5	1		5	1		5		1 (1.68 × 10^2^)
Liver lateral right	4	1	1 (8.19 × 10^2^)	5	1		5	1		5		1 (1.38 × 10^2^)
Liver median	3	2	1 (9.02 × 10^2^)	5	1		5	1		5		1 (1.32 × 10^2^)
Lung	4		2 (6.20 × 10^1^)	6			6			6		
Ovary	3									3		
Testis	3									3		

^a^BLD was defined as <12.5 copies/µg of DNA.

^b^BLQ was defined as <50 copies/µg of DNA.

^c^Quantity (copies/µg) of DNA for tissue samples and copy/µl for aqueous and vitreous humors.

^d^For lens samples, quantification of SPVN06 DNA (vector genome) was reported as copies/samples.

*BLD* below limit of detection, *BLQ* below lower limit of quantification, *LGN* lateral geniculate nucleus, *NHP* non-human primate, *RPE* retinal pigmented epithelium.

In the GLP low-dose study, SPVN06 DNA was also evaluated in NHP whole blood and tears before and after SPVN06 injection at a dose of 6.0 × 10^10^ vg/eye, which was the highest dose examined in the study (Table 4). No SPNV06 DNA was detected in blood or tear samples prior to dosing. Following injection, SPVN06 DNA was quantified in blood from Day 1 through Day 60/62 but became undetectable by Day 90. In tear samples, SPVN06 DNA was transiently quantified on Days 1 and 4, but not on subsequent days up to Day 90.

**Table 4:** Quantification of SPVN06 DNA in NHP whole blood and tears up to Week 13 after SPVN06 subretinal bilateral injection.

SPVN06 DNA (copies/µg DNA)	Week 13		
	6.0 × 10^10^ vg/eye		
	BLD^a^	BLQ^b^	Mean Copy^c^
*Blood*			
Predose	12		
Day 1			12 (8.69 × 10^3^)
Day 4			12 (6.26 × 10^3^)
Day 7			12 (1.09 × 10^3^)
Day 14	2	2	8 (7.02 × 10^2^)
Day 26/28	9	1	2 (6.73 × 10^2^)
Day 60/62	5		1 (1.32 × 10^2^)
Day 90	6		
*Tears Right*			
Predose	12		
Day 1			12 (1.50 × 10^6^)
Day 4	4	2	6 (5.43 × 10^3^)
Day 7	9	3	
Day 14	12		
Day 26/28	12		
Day 60/62	6		
Day 90	6		
*Tears Left*			
Predose	12		
Day 1			12 (1.73 × 10^6^)
Day 4		8	4 (5.42 × 10^3^)
Day 7	9	3	
Day 14	10	2	
Day 26/28	12		
Day 60/62	6		
Day 90	6		

^a^BLD was defined as <12.5 copies/µg of DNA.

^b^BLQ was defined as <50 copies/µg of DNA.

^c^Quantity in copies/µg of DNA.

*BLD* below limit of detection, *BLQ* below lower limit of quantification, *NHP* non-human primate.

### Ocular inflammation and immune response in NHPs

**Fig. 6:**
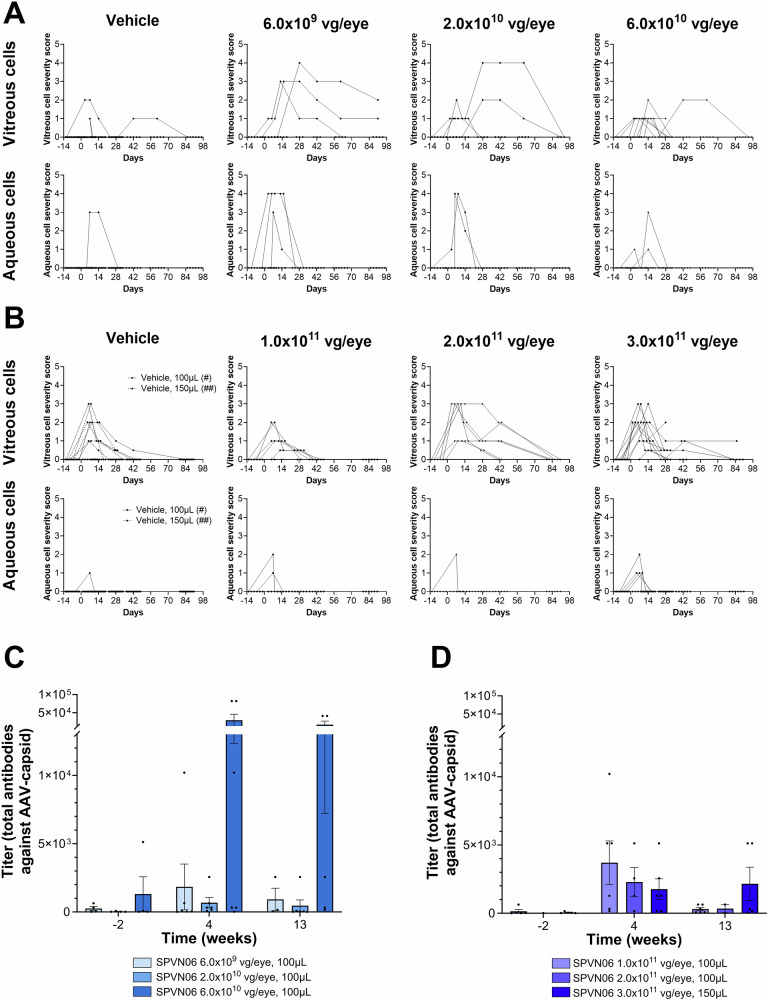
Ocular inflammation in NHPs following SPVN06 subretinal bilateral injection. Ocular inflammation in vitreous and aqueous cells in (**A**) GLP low-dose study and (**B**) GLP high-dose study. Ocular inflammation in individual animals was assessed before SPVN06 injection and up to Week 13 post-injection, using comparable standard grading systems for the number of cells observed in the field, based on the SPOTS system [25]. In the GLP low-dose study (**A**), a grading system that differentiated between aqueous and vitreous cells at the lower grades was used, with the following classifications: 0 (aqueous: 0, vitreous: ≤1 cell observed), 0.5 (aqueous: 1–5, vitreous: 2–5 cells observed), 1 (6–15 cells observed), 2 (16–25 cells observed), 3 (26–50 cells observed), and 4 ( > 50 cells observed). In the GLP high-dose study (**B**), the classification was the same for aqueous and vitreous cells, with grades defined as follows: 0 (0–5 cells observed), 1 (6–25 cells observed), 2 (26–50 cells observed), 3 (51–100 cells observed), and 4 ( > 100 cells observed). Each point represents the maximal grade from both eyes of an individual animal (*N* ≥ 6 in each dosing group). Total anti-AAV antibody serum titers before SPVN06 injection and up to Week 13 post-injection in (**C**) GLP low-dose study and (**D**) GLP high-dose study. Data shown are mean ± SEM (*N* = 6 animals per dose). AAV adeno-associated virus, GLP Good Laboratory Practices, NHP non-human primate, SPOTs Semiquantitative Preclinical Ocular Toxicology Scoring System. # vehicle control for 1.0 and 2.0 x 10^11^ vg/eye, ## vehicle control for 3.0 x 10^11^ vg/eye.

Ocular inflammation was assessed using a hand-held slit-lamp biomicroscope prior to injection with SPVN06 and on Days 3, 7, 14, 28, 42, 61, 85, and 91 post-injection in both NHP GLP studies. Transient inflammation, characterized by the presence of vitreous and aqueous cells, was observed in both vehicle- and SPVN06-treated NHPs (Fig. 6A, B). This procedure-related, non-adverse inflammation peaked at Week 1–2 post-injection and resolved by the end of the studies. Notably, no SPVN06 dose-response relationship was observed.

Immunogenicity was evaluated by measuring total anti-drug antibody levels in primate serum (Fig. 6C, D). Most animals exhibited low levels of pre-existing antibodies against the AAV8 capsid. In most SPVN06-treated animals from both GLP studies, antibody levels peaked at Week 4 post-injection and decreased to near baseline values by Week 13 post-injection, except in some animals treated at a dose of 6.0 × 10^10^ vg/eye, where antibody titers remained elevated at both Weeks 4 (3/6 animals) and 13 post-injection (2/6 animals). Antibody levels remained low in vehicle-treated animals, with no increase observed (data not shown). No total anti-transgene product antibodies were detected, and there was no T-cell-mediated systemic immune response against the capsid or transgenes (ELISpot) peptides at the highest SPVN06 dose tested (3.0 × 10^11^ vg/eye, data not shown).

### Retinal function in NHPs

**Fig. 7:**
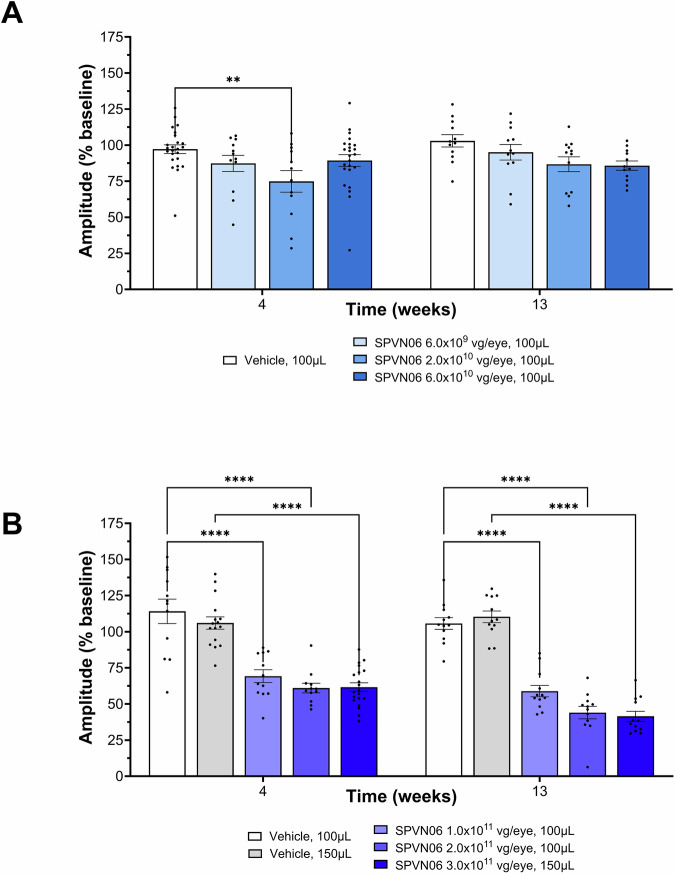
Retinal function in NHPs following SPVN06 subretinal bilateral injection. Retinal function in (**A**) GLP low-dose study and (**B**) GLP high-dose study was measured using ffERG, with photopic b-wave amplitude used as a measure of cone retinal function. A minimum of 12 and up to 24 individual eyes data were collected per dosing group and per timepoint, each dot represents individual data point. Statistical analysis was performed using a two-way ANOVA (Tukey’s post-hoc test), where **p ≤ 0.01 and ****p ≤ 0.0001. ffERG full-field electroretinography, NHP non-human primate.

Cone retinal function in NHPs following SPVN06 injection was assessed using ffERG photopic b-wave amplitude at Weeks 4 and 13 post-injection (Fig. 7A, B). Analyses revealed that at Week 13 post-injection, cone function was similar between animals treated with vehicle and those treated with SPVN06 at doses up to 6.0 × 10^10^ vg/eye (GLP low-dose study), indicating no significant adverse effects on cone function at these lower doses. Although there was a transient decrease in cone function in animals treated with SPVN06 at a dose of 2.0 × 10^10^ vg/eye, this resolved by Week 13. However, in animals treated with SPVN06 at higher doses (1.0–3.0 × 10^11^ vg/eye, GLP high-dose study), a significant dose-dependent decrease in cone function was observed at Week 4 compared with vehicle-treated animals (between −42% and −51%) and maintained through Week 13. This decrease in retina function for high doses was consistent with cone and RPE loss as described in the histology results section below.

### Histology in NHPs

The effects of SPVN06 at the tissue and cellular level were investigated using H&E staining in tissues collected at Weeks 4 and 13 post-injection. Drug-related microscopic findings were limited to the ocular tissues of animals administered SPVN06 at doses of ≥1.0 × 10^11^ vg/eye (GLP high-dose study). Modifications included loss of cone photoreceptors and RPE, which were confirmed by reductions in cone arrestin staining observed in both the peripheral and central retina, as well as in RPE65 staining of the peripheral RPE (Supplementary File 1). In the GLP low-dose study ( ≤ 6.0 × 10^10^ vg/eye), minor procedure-related findings were noted in the RPE of both vehicle- and SPVN06-treated animals at Week 4 post-injection, including slight hypertrophy and cellularity; however, these findings were not present at Week 13 post-injection. These minor procedure-related findings had no functional correlate and were fully resolved by Week 13.

## Discussion

RCD are progressive and degenerative diseases that ultimately results in blindness for many patients [1]. Due to the broad range of genetic mutations that cause RCD, there is a need for novel gene therapies that can slow retinal degeneration, regardless of the specific causative mutation [12]. Here, preclinical safety and biodistribution results, as well as pharmacological proof-of-concept, are reported for SPVN06, a gene- and mutation-agnostic AAV-based gene therapy that has the potential to preserve vision by slowing the degeneration of cone photoreceptors, irrespective of the underlying genetic mechanism or disease stage. Taken together, the findings support the ongoing clinical investigation of SPVN06.

All the studies reported herein were conducted in compliance with relevant regulatory guidelines [27–29]. The pharmacological activity of SPVN06 was evaluated in the *rd10/rd10* mouse model, which shares characteristics with the pathophysiology of human RCD (e.g. rod degeneration followed by cone death due to a lack of RdCVF and RdCVFL) and, to some extent, reflects the macroscopic and functional clinical features of the human disease [23, 30, 31]. SPVN06 at a dose of 1.0 × 10^8^ vg/eye was found to preserve visual function at all measured timepoints in comparison with *rd10/rd10* Vehicle-treated animals. These results are consistent with previous data from a pig model of RP, which demonstrated structural preservation of the retina after SPVN06 administration [32]. Based on mRNA transgene levels in the *rd10/rd10* retina, the pharmacologically active dose of 1.0 × 10^8^ vg/eye in mice was calculated to be equivalent to 5.0 × 10^9^ vg/eye in NHPs. Anatomical parameters were not considered in this calculation due to the limited ocular anatomical similarities between mice and NHPs.

NHPs were selected for safety and biodistribution studies due to their high degree of ocular anatomical similarity to humans, including the presence of a fovea and macula at the center of the retina [33, 34]. A subretinal route of administration was chosen for SPVN06 to ensure effective transduction of photoreceptors and RPE, providing extensive coverage of the retina. Importantly, RPE cells are expected to express and secrete the RdCVF transgene protein, which will be supplied to cone photoreceptors. Subretinal administration is used in approved therapies for a variety of ophthalmic conditions [35, 36]. This route of administration is clinically well-tolerated [37], as demonstrated by experience with the subretinal administration of voretigene neparvovec-rzyl (Luxturna^®^), which showed favorable safety outcomes in patients with retinal dystrophy caused by *RPE65* mutations [8, 9]. In addition, nonclinical data demonstrated that subretinal administration of SPVN06 was well tolerated in NHPs up to 6.0 × 10^10^ vg/eye, supporting the use of SPVN06 via the subretinal route of injection in the ongoing Phase I/II PRODYGY clinical trial (NCT05748873).

The present biodistribution study in NHPs demonstrated that SPVN06 DNA remained mostly localized to the retina and other ocular tissues following subretinal administration, with minimal distribution to systemic tissues. There were no SPVN06-related macroscopic or microscopic safety findings associated with the presence of SPVN06 DNA in systemic tissues. No SPVN06 DNA was detected in the reproductive system, reducing the risk of germline transmission. This distribution profile is consistent with the subretinal route of administration and aligns with previous studies of AAV8 subretinal injections in NHPs [26, 38, 39]. Transgene expression in the retina was maintained at steady levels until Week 13 post-injection, the last timepoint tested. Vector shedding in blood and tears was transient and low, consistent with previously reported results in both NHPs [26, 38, 39] and humans [40] receiving AAV8 injections.

Overall, the safety assessment in NHPs found SPVN06 to be well-tolerated up to a dose of 6.0 × 10^10^ vg/eye (GLP low-dose study). Transient procedure-related ocular inflammation was noted in both vehicle- and SPVN06-treated animals, with no apparent dose-response relationship. Similar findings were reported in a previous preclinical study, with transient presence of cellular infiltrate in the subretinal space at the site of AAV8 injection into NHP eyes [41]. In the present study, a transient and non-adverse immune response against the AAV capsid was noted, consistent with previous studies in NHPs [42].

For doses above 1.0 × 10^11^ vg/eye (GLP high-dose study), a significant decrease in cone retinal function was observed using ffERG, accompanied by corresponding histopathological changes, specifically affecting photoreceptors and RPE cells. Although toxicity associated with high AAV8 doses has been reported previously [41, 43], the specificity of the adverse effects observed may suggest minimal contribution from AAV8. Indeed, AAV8-related toxicity is generally associated with ocular inflammation [41], which was mostly absent in this program, even after the immunosuppressive regimen was discontinued. When ocular inflammation did occur, it was transient and attributed to the injection procedure. Supraphysiological levels of RdCVF and RdCVFL in a healthy NHP retina may offer a more likely explanation for the adverse effects observed at the highest doses examined. This phenomenon is not anticipated in RCD patients who exhibit a loss of rods and cones and thus a lack of endogenous RdCVF and RdCVFL proteins. RdCVF binds to basigin-1, forming a complex with glucose transporter 1 that increases glucose entry into cones, thereby promoting cone survival [15]. However, excess glucose entry can lead to cone-specific toxicity, as reported in a study in which oscillating hyperglycemia induced cone receptor dysfunction in zebrafish [44]. This was evidenced by prominent morphological degeneration and dysfunctional cone-mediated electroretinograms, independent of vascular changes. RdCVFL is a potent antioxidant, and while no reports exist on the specific toxicity of RdCVFL, overexpression of antioxidant enzymes can disrupt intracellular redox balance, potentially causing adverse effects [45].

A limitation of this preclinical program is the lack of an assay distinguishing endogenous from exogenous RdCVF and RdCVFL at the protein level, complicating the interpretation of the adverse effects observed at high doses of SPVN06. While mRNA analyses can differentiate *RdCVF* and *RdCVFL* transgenes via unique nucleotide sequences, the RdCVF and RdCVFL proteins produced by SPVN06 are identical to human endogenous proteins; thus, establishing protein-level methods in NHPs is difficult due to the high conservation of these proteins across species. In mice, RdCVF, RdCVFL, and basigin-1 share ~97%, 86% and 80% sequence similarity with human proteins, respectively, while NHPs exhibit even higher similarity (99%, 99% and 89%, respectively). In humans, quantifying these proteins is particularly challenging, as retinal biopsies cannot be performed in living patients. While access to postmortem human retina is limited, RdCVFL is intracellular and RdCVF is secreted, further complicating quantification. Therefore, even if NHP data were available, translating these results would remain challenging, especially in the context of disease.

Beyond sequence similarity, several additional considerations guided the selection of animal models for this preclinical program. As previously mentioned, the *rd10/rd10* mouse mirrors important aspects of the pathophysiology of human RCD, including the progressive rod degeneration, followed by cone death, due to the lack of RdCVF [23, 30, 31]. In addition, the ability of AAV8 to transduce photoreceptors and RPE in *rd10/rd10* mice following subretinal injection has been demonstrated previously [46]. Given the pronounced decline in ffERG and OKT observed in *rd10/rd10* mice during disease progression, functional endpoints can be effectively investigated. However, the accelerated progression of degeneration in these mice (over weeks), compared with the more gradual course in humans (over decades), limits the direct translation of results from this model to humans in terms of the durability of the biological response. However, as long-term expression of RdCVF and RdCVFL is expected in cones and RPE cells, which are non-dividing, the protective effect of SPVN06 is anticipated to be long-lasting. NHPs were selected for safety and biodistribution studies not only due to their ocular anatomy, which closely resembles that of humans, but also the ability of AAV8 to transduce primate retinal cells following subretinal delivery [47]. This allowed for the investigation of the device and route of administration used in clinical development. With normal retinal structure and normal endogenous levels of RdCVF and RdCVFL, healthy NHPs provide a highly relevant system for SPVN06 safety and biodistribution assessment. Although large animal models of RCD exist, such as the TgP23H hRHO pig, they were not selected for a hybrid pharmacology and safety study due to limited functional and phenotypic characterization to inform safety. In summary, the preclinical program for SPVN06 fulfilled its primary purpose of informing and supporting the clinical development of SPVN06.

Based on the results of the second 13-week NHP study evaluating SPVN06 doses ranging from 6.0 × 10^9^ vg/eye to 6.0 × 10^10^ vg/eye (GLP low-dose study), the no observed adverse event level (NOAEL) was determined to be 6.0 × 10^10^ vg/eye. Considering the differences in retinal surface area between NHPs and humans (2-fold larger in humans), the equivalent dose of 6.0 × 10^10^ vg/eye was estimated to be 1.2 × 10^11^ vg/eye in humans. The selection of a clinical starting dose is then based on the nature of the safety findings in NHPs while considering appropriate safety margins.

In conclusion, SPVN06 was pharmacologically active in the *rd10/rd10* mouse model and well-tolerated in NHPs at doses up to 6.0 × 10^10^ vg/eye. The SPVN06 nonclinical package supports the continued investigation of SPVN06 in the ongoing clinical Phase I/II trial, PRODYGY (NCT05748873). A total of 33 patients will be enrolled in the trial and followed for up to 5 years. Safety data in the Phase I dose-escalation of PRODYGY, including 1.5 years of follow-up for the low-dose cohort, 1 year for the medium-dose cohort, and between 3 and 9 months for the high-dose cohort, have demonstrated a favorable safety profile [20]. Patient enrollment in the Phase II extension is ongoing [19].

## Supplementary information

Supplementary Files

## Data Availability

The datasets generated during and/or analyzed during the current study are not publicly available due to commercial and legal concerns but are available from the corresponding author upon reasonable request.
