# Supplementary material for: Preclinical safety and biodistribution of SPVN06, a novel gene- and mutation-independent gene therapy for rod-cone dystrophies
Source: Gene Ther. 2025 Aug 4;33(4):421–34. doi: 10.1038/s41434-025-00556-3 (PMC13473012; doi:10.1038/s41434-025-00556-3)

Supplementary files and legends

Supplementary File 1: Retinal histology after SPVN06 subretinal injection in NHPs.

Representative images of paraffin retinal sections after hematoxylin and eosin (H&E) staining 1 and 3 months after SPVN06 subretinal injection at (A) 6.0×10^{10} and (B) 3.0×10^{11} vg/eye. Immunohistochemistry with cone (cone arrestin) and RPE (RPE65) specific markers. (A) No changes were observed at the SPVN06 dose of 6.0×10^{10} vg/eye in comparison to Vehicle-treated eye. (B) Decreases in cone arrestin with loss of cone cell population as well as a decrease in RPE65 staining was observed at the highest SPVN06 dose of 3.0×10^{11} vg/eye.

Supplementary File 2: ffERG, OCT and histological evaluation of SPVN06 in the rd10/rd10 mouse model of RCD.

(A-C) There was no significant slowing of visual function loss measured by ffERG ($N \geq 20$ eyes/group). (D and E) There was no obvious structural protection of the retina on the OCT imaging measurements. (F and G) Illustrative images of hematoxylin and eosin histology (P48) revealed comparable retinal anatomy with one row of nuclei in the ONL in both vehicle and SPVN06-treated retina (1.0×10^{8} vg/eye) as showed by cone arrestin staining in pink (B). P: Postnatal day, RGC: retinal ganglion cells, IPL: inner plexiform layer, INL: inner nuclear layer, ONL: outer nuclear layer, RPE: retinal pigment epithelium.

Supplementary File 1

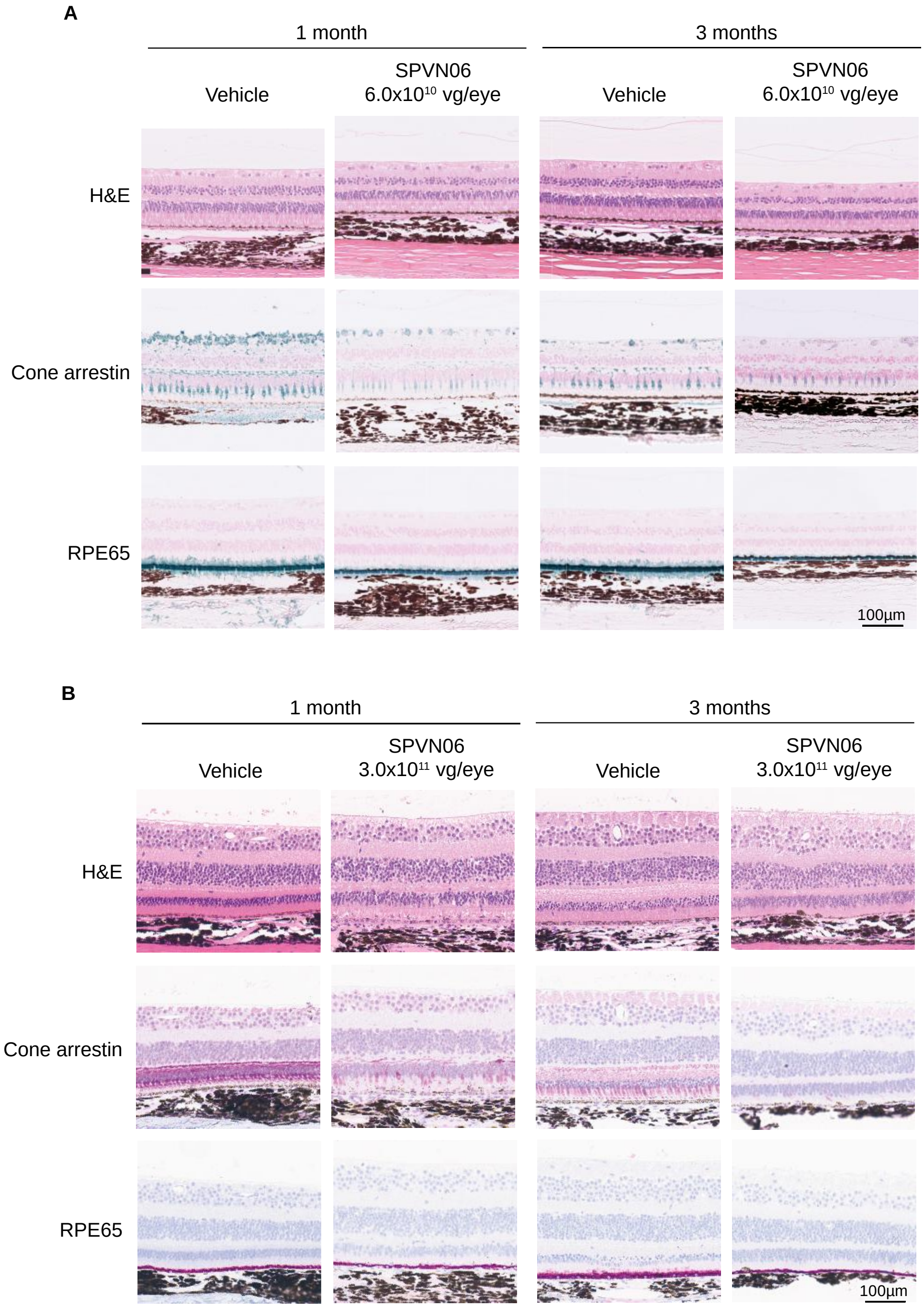

Supplementary File 2

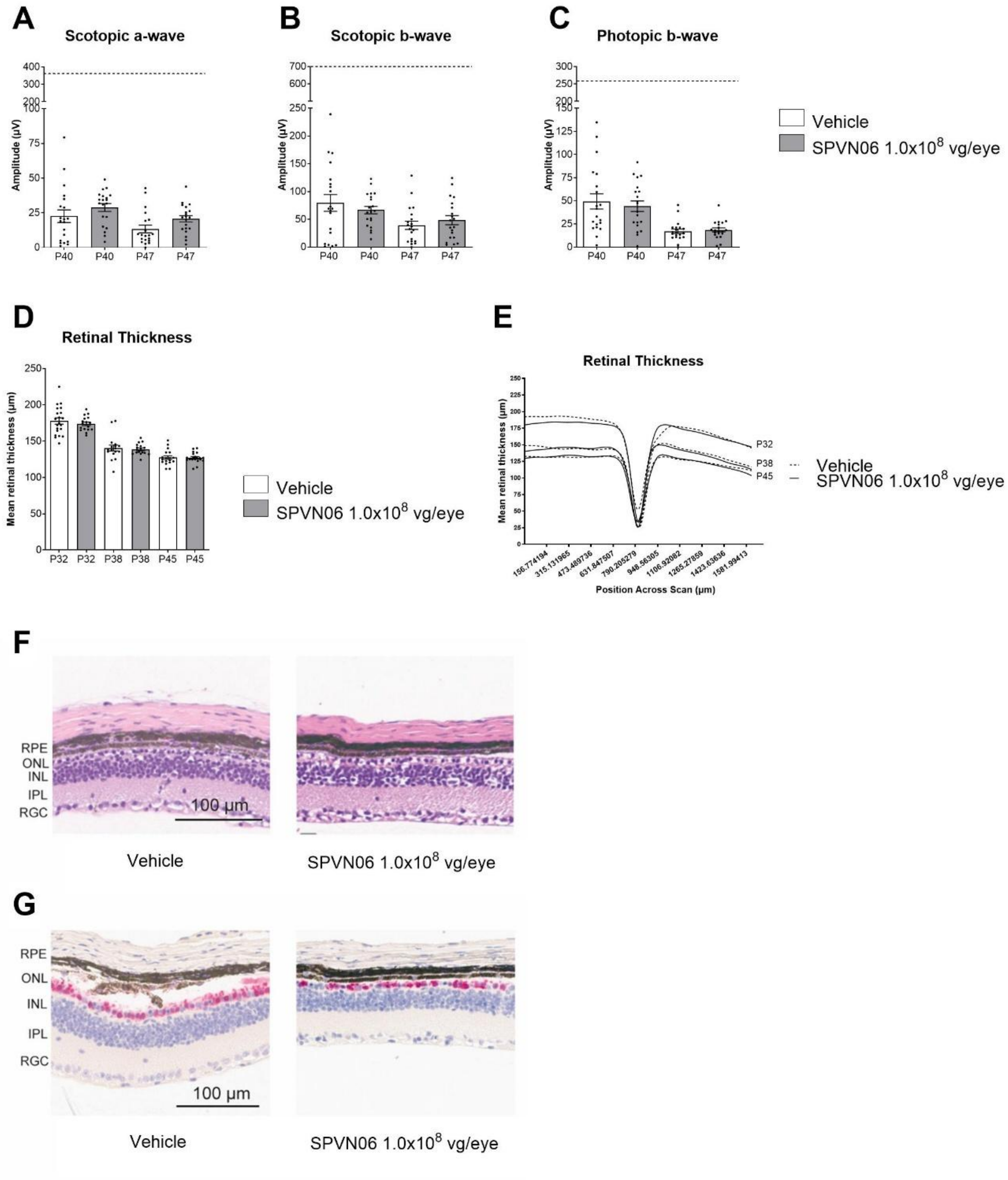

Supplement: Supplementary file 1 [file 41434_2025_556_MOESM1_ESM.pdf]
